# The Demand–Control Model and Pupils’ Aggressive Behaviour towards Teachers: A Follow-Up Study

**DOI:** 10.3390/ijerph181910513

**Published:** 2021-10-07

**Authors:** Lars Peter Andersen, Birgit Aust, Trine Nøhr Winding

**Affiliations:** 1 Danish Ramazzini Centre, Department of Occupational Medicine—University Research Clinic, Regional Hospital West Jutland, 7400 Herning, Denmark; trinwind@rm.dk; 2 National Research Centre for the Working Environment, 2100 Copenhagen, Denmark; BMA@nfa.dk

**Keywords:** teachers, demand–control model, pupils’ aggression

## Abstract

Purpose: Pupils’ aggressive behaviour towards teachers is a common phenomenon in schools across different countries. The purpose of this study is to test hypotheses that are central to the Job Demand–Control model as risk factors for pupils’ aggressive behaviour towards teachers. Method: Questionnaire data were collected in 2018 and 2019 from teachers at 94 public schools in Denmark. In total, 1198 teachers participated in both rounds. Demands and social support at work were measured in 2018, and pupils’ aggressive behaviour was measured in 2019. The analyses were performed using multivariate logistic regression analysis. Results: Teachers were often exposed to pupils’ aggressive behaviour during their work. High emotional work demands and low control were associated with increased risk of pupils’ aggressive behaviour. No mitigating effect of high control was found on the association between emotional demands and risk for pupils’ aggressive behaviour towards teachers. Conclusion: High emotional demands were strongly associated with the aggressive behaviour of pupils towards teachers. Job control over own work situation was not enough to lower the risk of aggressive behaviour under conditions in which teachers experience high emotional demands. Based on these results, we recommend that supervisors carefully balance teachers’ emotional demands to their resources.

## 1. Introduction

Since the introduction of the Job Demand–Control model by Karasek and Theorell [1], many scholars have sought to apply the model to a wide variety of stress reactions, which are mostly indicators of poor physical health or general and job-related ill-being. [2,3]. However, only few studies have examined social behavioural outcomes such as workplace bullying [4,5,6] or work-related violence towards employees [7]. Therefore, our aim is to test hypotheses that are central to the Job Demand–Control model in relation to work-related threats and violence towards teachers in public schools.

### 1.1. Pupils’ Aggressive Behavior towards Teachers

Pupils’ aggressive behaviour towards teachers is a common phenomenon in schools across different countries [8]. Despite this widespread problem, it is important to be aware of that most student aggression towards teachers is not only physical but often verbal [9]. However, due to variations in a number of aspects—for example, the way violence is defined (e.g., only physical violence or also non-physical violence), the timeframe for the assessment (e.g., in the last month or in the last year), or the age of the students (e.g., only younger children or all age groups), the results from different studies can be quite different. To address this problem, a recent meta-analysis calculated the prevalence across 24 studies from around the world and found a pooled prevalence of 53% for any type of pupils’ aggressive behaviour directed towards teachers reported over the last two years, although the prevalence ranged from 20% to 75% depending on the study [10].

Since studies use very different measurements and definitions, it is often misleading to compare the results of single studies directly with each other. Repeating the same measurements in the same population can give more reliable results about the prevalence and the development of the problem.

Pupils’ aggressive behaviour towards teachers often requires interaction of great emotional intensity [11] and the experience of pupils’ aggressive behaviour is found to have a negative impact on the general well-being, burn-out, performance, mental health of teachers and intention to leave the teaching profession [12,13,14,15,16].

The reasons for students’ aggressive behaviour are complex and often have their roots in frustrations or fear that escalate into aggressive behaviour if not understood and dealt with [17]. A recent study showed that especially students receiving special education services to a higher degree threaten teachers than students not receiving special education services. As the authors point out, an explanation might be that aggressive behaviour is the result of a student’s difficulty dealing with frustration [18].

However, there is no single explanation for the reason why teachers may be exposed to violence and threats of violence at work, as violence and threats of violence occur in an interpersonal context in which there will be factors in the environment that either inhibit or support the development of aggressive behaviour [19]. Although pupils’ aggression towards teachers in schools is a rather common phenomenon and the health consequences following work-related threats and violence may be serious, knowledge about work-environmental risk factors is limited. Studies from the US have found that teachers employed in urban schools are more often exposed to violence and threats than teachers in suburban schools [20,21]. However, these findings might not apply to other countries with different social structures and welfare systems. In a Danish study about the aggressive behaviour of pupils against teachers, no difference between schools in more rural and more urban settings were found [22]. Studies have found that consistent enforcement of school rules may reduce rates of pupils’ aggressive behaviour as well as an authoritative school environment may help reduce frequency [23,24,25]. An authoritative school climate can be described as being strict but fair with regard to disciplinary practices and to be characterised by supportive teacher–student relationships [18]. Additionally, high demands and low job control for teachers are found to be associated with increased risk of pupils’ aggressive behaviour [26].

To better understand the specific working conditions that increase the risk of pupils’ aggressive behaviour towards teachers, it might be helpful to use a theory-driven model such as the Job Demand–Control model (JDC model) [1]. The integration of the model into research about risk factors for work-related threats and violence is supported by theoretical arguments and empirical findings. Empirical studies have shown positive associations between high work demands, low influence over own work situation, and work-related violence [7,27,28]. This is also in line with the theoretical assumptions of the model stating that situations of high job demand and low job control may increase work-related strain in employees [29], which again may increase the risk of errors, mistakes, or blunders [4]. In turn, pupils may react with feelings of anger, frustration, as well as violent and threatening behaviour when teachers make errors or mistakes. 

### 1.2. The Job Demand–Control Model

The Job Demand–Control model (JDC) distinguishes between two crucial aspects of the job: namely, psychological demands and job control. Job demands refer to work load, work efforts, unexpected tasks, and conflicting demands. The second aspect of the model is job control, which refers to someone’s ability to control their own work situation, opportunity to organise their own work, adopt their own initiatives, experience autonomy, and have skill discretion, for example to learn new things and develop new competencies [30]. High job control gives the employees the opportunity to have decisional authority on how they handle the required work as well as to restrict the pacing, the timing, and the methods used in accomplishing the work tasks [29]. Two hypotheses have been derived based on the model: (a) the strain hypothesis, which states that the highest level of ill health is expected when the job is characterised by high job demands and low control at the same time (main effects), and (b) the buffer hypothesis, which predicts that high control can moderate the potential negative effects of high job demands on health. Thus, high job control is expected to moderate/buffer the negative effects of high job demands on employee health and well-being. A study found that teachers experiencing high demands and low decision authority were significantly more emotionally exhausted, had significantly lower scores in vitality and emotional well-being, and spent more days on sick leave during the last school term. Furthermore, in the study, high demands and low control were found to interact and together increase the risk of emotional exhaustion [31]. 

Many studies have applied the JDC model to a broad array of outcomes. Examples are heart diseases [32], depression [2], burnout [3] and well-being [33]. However, evidence for interactive effects as predicted by the buffer hypotheses of the JDC model is overall very weak [33].

### 1.3. The Demand–Control Model and Behavioural Outcome

Still, only few studies have highlighted the JDC model’s added value in explaining bullying and aggressive behaviour towards employees at work as a form of social behavioural outcomes [4,6,7]. Some studies have found that high demands and low control are associated with being a victim of bullying and that high job control can moderate such an association [4,34]. However, only few studies have used the JDC model to explore the relationship between the work demands and job control and aggressive behaviour towards employees perpetrated by clients, pupils, patients, and the like [7,27,28]. For instance, cross-sectional studies have applied the model among nurses and midwifes and found that high demands were a significant predictor of threats and emotional abuse; however, job control was not found to be associated with work-related violence or threats, and no moderating effects were observed [27,28]. Furthermore, among teachers, a cross-sectional study found that higher work demands were associated with the most offensive types of aggression, whereas control was only associated with property offenses and physical attacks [26]. In the human service sector, one prospective study found that high work demands and low job control were associated with increased risk of aggressive behaviour towards employees, but no moderating effects between high demands and low job control were found [7].

In additional to the type of critique presented in the precious paragraph, even though these studies were carried out in the human service sectors, demands were only conceptualised as quantitative demands, which may have led to misinterpretations of the model’s assumptions, because since the model was developed by Karasek and Theorell [1], the nature of job demands has changed considerably as a consequence of the changing nature of work in the Western world [35]. Thus, for many employees, there has been a shift from physical demands to mental demands [36] and emotional demands [37]. Therefore, in the case of human service work including teaching, there is a need to update the Job Demand model. For this purpose, it has been suggested to include emotional demands and thereby focus on demands that might exist when interacting with clients, patients, or pupils [29]. Several researchers have demonstrated the importance of including emotional demands in the Demand–Control model [38,39]. Söderfeldt et al. found that increased quantitative demands was related to decreased psychological symptoms, whereas increased emotional demands was related to increased psychological symptoms. Thus, only considering the quantitative demands would have led to misinterpretations of the JDC model. Against this background, the authors claimed that their results emphasise the specificity when measuring human service jobs and that human service work is emotionally taxing [38]. 

Being a teacher is often described as an occupation with high work demands. Studies have found that teachers often report increased work pace, concerns about classroom discipline, and high demands at work, especially emotional demands [40,41,42,43]. In order to be a good teacher, it is important to be able to draw students’ attention and motivate the pupils. Furthermore, as caregivers in the absence of their parents, teachers must support the pupils and be attuned to their needs and function [44].

Against this background, the present article therefore explores whether the JDC model’s strain hypothesis can be applied to target’s reports of pupils’ aggressive behaviour as a new outcome variable. More specifically, we will use the JDC model to explore the relationship between teachers’ emotional demands and their influence over their own work situation and the risk of pupils’ aggressive behaviour towards teachers in public schools. 

The study has the following aims:

1. To examine if high levels of emotional demands at work are related to increased reporting of pupils’ aggressive behaviour towards teachers.
2. To examine if low levels of influence over own work situation is related to increased reporting of pupils’ aggressive behaviour towards teachers.
3. The examine if high emotional demands at work and pupils’ aggressive behaviour towards teachers is mitigated by high levels of influence over own work situation, i.e., if the association decreases when the influence is high.

## 2. Methods

### 2.1. Study Population

The study sample consists of a cohort of school teachers from municipal primary and lower secondary schools (grades 0 to 9) in Denmark. From all municipal schools of this type in Denmark, we selected both small and large schools as well as schools from all five main regions of Denmark. Only teachers were invited in this study (e.g., no pupils participated).

The baseline survey was conducted between September and December 2018. Altogether, 4935 teachers from 105 schools were invited to participate. The teachers’ e-mails were obtained from the participating schools, and two consecutive questionnaires were sent with 1-year time interval. Participation in the study was voluntary, and collected data were treated confidentially. Initially, 2336 teachers participated. At follow-up, 94 of the original 105 invited schools agreed to participate, resulting in 1830 potential participants. The final study population for the present study included teachers having answered both baseline as well as the follow-up questionnaires (*n* = 1.198). 

### 2.2. Exposure

#### 2.2.1. Emotional Demands at Work

The job demand component of the JDC model was operationalised in terms of emotional demands at work. The latter was measured with a scale from the Danish Psychosocial Work Environment Questionnaire (DPQ), which is a standardised instrument designed to measure different aspects of the psychosocial work environment [45].

The scale for emotional demands includes three items (e.g., ‘Does your job put you in emotionally demanding situations?’; ‘In your job, do you have contact with pupils, parents, or others who are reluctant or aggressive towards you?’; ‘Do you have relationships in your job that are emotionally difficult to deal with?’) to be answered using five Likert-type response options, ranging from ‘always’ (100) to ‘never’ (0). The scale was dichotomised into high emotional demands (≥75) and low emotional demands (<75).

Cronbach’s alpha for this scale was 0.77.

#### 2.2.2. Influence over Own Work Situation

Control over one’s own work situation was measured by the ‘influence over own work’ scale from the Danish Psychosocial Work Environment Questionnaire (DPQ) [45].

The scale includes two items (‘Do you have influence on how to solve your work tasks?’ and ‘Do you have opportunities to make significant decisions regarding your job’?) to be answered using five Likert-type options, ranging from ’always’ (100) to ‘never’ (0). The scale was dichotomised into high influence over one’s own work situation (≥75) and low influence over one’s own work situation (<75).

Cronbach’s alpha for this scale was 0.81.

#### 2.2.3. Outcome—Pupils’ Aggressive Behavior

Information about pupils’ aggressive behavior was obtained from the follow-up questionnaire in 2019.

Based on the definition by Wynne, Clarkin, Cox, and Griffiths [46]), we applied a broad definition of aggressive behaviour at work including (1) harassment, (2) threats, and (3) violence against teachers. All items were measured on a five-point Likert-scale ranging from never (0) to daily (4) [46]. 

Harassment was measured by 10 items (e.g., How often during the last 12 months have you at your school been exposed to the following which is related to harassment: being called degrading things, being verbally patronised, etc.). A total sum score with a possible range of 0–40 was calculated for each participant, and the scale was then dichotomised at the 75th percentile into high level of harassment (≥6) or low level of harassment (≤5). Cronbach’s alpha for this scale was 0.77

Threats were measured by 7 items (e.g., How often have you at your school during the last 12 months been exposed to the following which is related to threats: being threatened with objects, being threatened with beatings etc.). The perpetrator could be pupils or parents. A total sum score with a possible range of 0–28 was calculated for each participant and the scale was then dichotomised at the 75th percentile into high level of threats (≥2) or low level of threats (≤1). Cronbach’s alpha for this scale was 0.74.

Violence was measured by 12 items (e.g., How often have you at your school during the last 12 months been exposed to the following which is related to violence: being hit, hit with an object, scratched/pinched, etc.). The perpetrator could be pupils or parents. A total sum score with a possible range of 0–48 was calculated for each participant, and the scale was then dichotomised at the 75th percentile into high level of violence (≥3) or low level of violence (≤2). Cronbach’s alpha for this scale was 0.92.

The threats and violence scales have been used in other studies carried out in others sectors [47,48,49]. Factor analysis (direct oblimin; eigenvalue = 1) carried out in this study population showed that the threats and violence scale each consisted of one dimension (results not shown).

### 2.3. Confounders

Information about age and gender was derived from the baseline questionnaire in 2019.

### 2.4. Statistical Analysis 

Baseline population characteristics were calculated for all outcomes, exposures, and potential confounders as number and percentile distribution. Since data did not meet the statistical assumptions of normality and homoscedasticity required for linear regressions [50], multilevel logistic regression was conducted in order to take into account the possible correlations between teachers from the same schools. Considering the range of different school sizes and the large numbers of schools (N = 94), using school-level predictors is considered to be minimally biased [51,52]. Crude and adjusted odds ratios (ORs) with 95% confidence intervals (CIs) were conducted for the associations between emotional demands and influence over own work situation and the three types of pupils’ aggressive behaviour (harassment, threats, and violence) 1 year later (i.e., taken from the follow-up survey), taking into account potential confounders (i.e., taken from the baseline survey). Subsequently, the associations between emotional demands and the three types of pupils’ aggressive behaviour were stratified for influence over own work situation. All statistical analyses were performed using the statistical software package SPSS, version 22 (IBM Corp, Armonk, NY, USA).

## 3. Results

Table 1 shows the descriptive statistics of the sample. Almost three-quarters of the participants were women (74%). Most participants were in the age groups 41–50 years (37%) and 51–60 years (26%), while a few participants were between 21 and 30 years of age (7%). More than half of the participants had seniority between 6 and 20 years (52%). 

Responders and non-responders at follow-up were compared according to gender, age distribution, and seniority. No difference according to gender was found. However, slightly more teachers in the age groups ≤ 21–30 and >60 were among non-responders compared to responders (max. 6% difference in the age groups) and slightly more teachers with ≤5 years and >20 years of work experience were found among the non-responders compared to responders (max. 8% difference in the work experience groups).

The means, standard deviations, and correlations of study variables are presented in Table 2. It can be seen that emotional demands are positively correlated with higher levels of reported harassment, threats, and violence. Regarding influence over own work situation, it can be seen that harassment, threats, and violence is negatively correlated with reported levels of influence over own work situation.

From Table 3, it can be seen that high emotional demands are statistically significantly associated with all three types of pupils’ aggressive behaviour after adjusting for gender, age, and baseline aggressive behaviour.

From Table 4, it can be seen that the association between influence over own work situation and pupils’ aggressive behaviour is mixed. Low levels of influence over own work situation statistically significantly increased the risk of reporting harassment and threats, but for threats only in the unadjusted model. Furthermore, low influence over own work situation slightly decreased the risk of reporting physical violence in both the unadjusted model in the adjusted model, both showing statistically non-significant estimates.

Finally, Table 5 shows the associations between emotional demands and pupils’ aggressive behaviour stratified by high and low levels of influence over own work situation. It can be seen that regarding the associations between high emotional demands and the three types of pupils’ aggressive behaviour in case of high influence over own work situation, the ORs were between 1.53 and 1.62 which is very similar to the non-stratified results in Table 3 (adjusted models). Furthermore, from Table 5 it can be seen that low levels of influence over own work situation increase the risk for threats whereas low level of influence decreased the risk for violence in case of high emotional demands.However, the results are not statistical significant. 

## 4. Discussion

The current study set out to empirically examine if the JDC model could be predictive of pupils’ aggressive behaviour towards teachers in form of work-related harassment, threats, and violence. Even though we dichotomised the outcomes at the 75th percentile and labelled this high level of harassment, threats, and violence, the mean values for harassment, threats, and violence tend to be rather low, which means that episodes of harassment, threats, and violence occur relative rarely. 

We found that high emotional demands were related to increased risk of all three types of pupils’ aggressive behaviour, and low influence over own work situation was associated with increased risk of harassment and threats. However, no mitigating effect of high influence over own work situation on the association between emotional demands and pupils’ aggressive behaviour was found, which means that emotional demands increase the risk of pupils’ aggressive behaviour irrespective of the level of influence over own work situation. This is in line with a previous review concluding that the evidence of mitigating effects, as predicted by the buffer hypotheses of the Job Demand–Control model, is very weak [33]. 

Previous studies have found that high demands and low job control are associated with increased risk of teachers being exposed to pupils’ aggressive behaviour [26,53]. However, to capture the specific demands among teachers, the specific context of teaching must be taken into account. Several studies have found that teachers often report high emotional demands [40,41,42,54,55,56]. Teaching requires careful management and control of emotional expressions during interactions with pupils [57,58,59,60], because teachers are frequently exposed to challenging situations such as conflicts, misbehaviour, and aggressive behaviour [10,61,62,63]. However, even if the pupils’ misbehaviour or aggressive behaviour arouses anger or anxiety in teachers, the teacher–pupil interaction requires that teachers control their emotional expressions [64,65]. Thus, due to the complexity of the teacher–student relationship, teachers need to engage in an extensive degree of emotionally demanding work [56,66].

Therefore, for both scientific and practical reasons, it is important to include specific measures of job demands to assess the job context, thereby increasing the ecological validity of the assessment of the work environment and possible associations with different outcomes. Thus, the assessment of specific job demands is beneficial to the development of tailored occupational interventions [30]. 

The study supports the notion that an adverse psychosocial work environment may act as fertile ground for the occurrence of aggressive behaviour at work [7,27,28]. More specifically, we found that high emotional demands and low influence over own work situation both increase the risk of pupils’ aggressive behaviour. One explanation may be that high levels of emotional demands wear out teachers’ resources and lead to difficulties in coping with potential aggressive behaviour from pupils. Furthermore, in the literature, influence over own work situation is viewed as a central resource that can improves the employees’ capacity to make decisions [29]. Therefore, teachers’ high influence over own work situation may potentially act as a resource giving possibilities for teachers to deliver the best didactic method to ensure pupils’ engagement, as pointed out by Lamb and Reinders [67]. 

Indirectly, this is supported by our study as we found that low influence increased the risk of work-related harassment, threats, and violence. The mechanism might be that low influence over own work situation decreases the possibilities to make the right didactic decisions and thus can be an obstacle to deliver teaching in a way that could reduce the risk for work-related harassment, threats, and violence. 

We did not find any mitigating effect of high influence over own work situation on the association between high emotional demands and pupils’ aggressive behaviour. The result is somewhat surprising, because according to Hackman and Oldham’s Job Characteristics Model [68], influence over own work situation is expected to result in increased motivation and effectiveness, because higher influence leads to greater confidence in performing the tasks [69]. One explanation for the lacking mitigating effect of influence might be a mismatch between the items used to measure influence/control and the teachers’ emotional demands. Job control might have to correspond better to the emotional demands on teachers in schools to mitigate the impact of high emotional demands on pupils’ aggressive behaviour [29]. A further explanation is that the Demand–Control model is too narrow in its focus [70], only taking a minor part of the work environment into account. It has been pointed out that employees are not only influenced by the nature of the work tasks but also influenced by social relations at work as well as the contextual systems in which the employees are embedded [71]. 

Another explanation as to why influence over own work situation did not decrease the risk of pupils’ aggressive behaviour under the condition of high emotional demands may be the teachers’ lack of the right competencies to apply high influence to decrease pupils’ aggressive behaviour under the condition of high emotional demands. If the teachers assessed themselves as lacking the right competencies in relation to the responsibility that follows with influence over own work situation, they may feel demotivated to engage in decreasing the pupils’ aggressive behaviour under the condition of high emotional demands [72]. According to the Motivated Competence Model, a sufficient degree of influence as well as competencies are necessary for the best performance [73]. However, over the past decade, pupils with developmental disorders have been included in the ordinary classes, and they require certain competencies to teach [74]. The question is whether the teachers feel they have the competencies to teach and handle these pupils in ordinary classes, which is a topic that often has been discussed [75]. Thus, even though teachers have influence over their own work situation, they may lack the right competencies to apply this influence to decrease the risk for pupils’ aggressive behaviour under the condition of high emotional demands.

## 5. Strengths and Limitations

The present study has some important strengths. The study is based on a longitudinal design including different types of public schools (small, large, located in both small and big cities), and the sample is rather large. Furthermore, the distribution of gender and age in the study population is corresponding to the members of the Danish Teacher Association (96% of all teachers in Denmark are members).

Yet, the results of the present study should be considered in the light of some limitations as well. Even though the total sample size was rather large, the schools were recruited using a non-random sampling method. Furthermore, several schools refused to participate, so we cannot rule out some potential selection bias, which may reduce the external validity of the findings. Third, the data were entirely based on self-reports, which may introduce mono-method bias due to unmeasured third variables [76]. Finally, a better understanding of the context and complexity of these associations would require more in-depth qualitative studies, because the survey questions only give us some more general information.

## 6. Conclusions

We found that high emotional demands increased the risk of pupils’ aggressive behaviour in the form of harassment, threats, and violence and that low influence over one’s own work situation increased the risk of pupils’ aggressive behaviour. However, under conditions of high emotional demands, influence over own work situation did not decrease the risk of pupils’ aggressive behaviour. 

Thus, despite the fact that influence over one’s own work situation can decrease the risk of aggressive behaviour from pupils towards teachers, influence over one’s own work situation is not enough to lower the risk of aggressive behaviour under conditions in which teachers experience high emotional demands. Based on these results, we therefore recommend that supervisors carefully balance teachers’ emotional demands to their resources.

## Figures and Tables

**Table 1:** Population characteristics, n = 1198.

	n (%)
Gender	
women	888 (74)
men	296 (25)
missing	14 (1)
Age	
≤21–30	79 (7)
≤31–40	241 (20)
≤41–50	445 (37)
≤51–60	315 (26)
>60	98 (8)
missing	20 (2)
Seniority	
≤5 years	151 (13)
6-20 years	624 (52)
>20 years	405 (34)
missing	18 (2)
Emotional demands at work, baseline *	
Low	918 (77)
High	256 (21)
Missing	24 (2)
Influence at baseline *	
Low	302 (25)
High	885 (74)
Missing	5 (0.4)
Harassment, follow-up *	
low	983 (74)
high	333 (25)
missing	6 (0.5)
Threats, follow-up *	
low	971 (73)
high	346 (26)
missing	5 (0.4)
Violence, follow-up *	
low	967 (73)
high	349 (26)
missing	6 (0.5)

* cut off at the 75th percentile.

**Table 2:** Mean, standard deviation, and correlation of variables.

	Mean	SD	1	2	3	4	5	6	7	8
1. Emotional demands	149.02	51.46	1	−0.15 *	0.48 *	0.40 *	0.34 *	0.36 *	0.31 *	0.29 *
2. Influence over own work situation	144.31	34.20	−0.15 *	2	−0.13 *	−0.05	−0.01	−0.16	−0.08	0.01
3. Exposed to harassment at baseline	4.30	4.10	0.48 *	−0.13 *	3	0.69 *	0.62 *	0.58 *	0.49 *	0.44 *
4. Exposed to threats at baseline	1.32	2.30	0.40 *	−0.05	0.69 *	4	0.75 *	0.44 *	0.56 *	0.51 *
5. Exposed to violence at baseline	2.52	4.68	0.34 *	−0.01	0.62 *	0.75 *	5	0.44 *	0.54 *	0.61 *
6. Exposed to harassment at follow-up	4.16	4.24	0.36 *	−0.16 *	0.58 *	0.44 *	0.44 *	6	0.72 *	0.62 *
7. Exposed to threats at follow-up	1.25	2.30	0.31 *	−0.08 *	0.49 *	0.55 *	0.58 *	0.72 *	7	0.75 *
8. Exposed to violence at follow-up	2.39	4.86	0.29 *	0.01	0.44 *	0.51 *	0.61 *	0.62 *	0.75 *	8

* Correlation is significant at the 0.01 level (2-tailed).

**Table 3:** Associations between emotional demands at work and harassment, threats and violence.

	Harassment		Threats		Physical violence	
	Unadjusted	Adjusted **	Unadjusted	Adjusted **	Unadjusted	Adjusted **
	OR (Cl)					
Low emotional demands (ref)	1	1	1	1	1	1
High emotional demands *	3.26 (2.42–4.41)	1.50 (1.04–2.15)	3.21 (2.37–4.34)	1.53 (1.06–2.21)	2.96 (2.17–4.03)	1.51 (1.03–2.23)

* cut off at the 75th percentile, ** Adjusted for gender, age and baseline harassement, threats or violence.

**Table 4:** Associations between influence over own work situation and harassment, threats and violence.

	Harassment		Threats		Physical violence	
	Unadjusted	Adjusted **	Unadjusted	Adjusted **	Unadjusted	Adjusted **
High influence (ref)	1	1	1	1	1	1
Low influence *	1.97 (1.41–2.78)	1.78 (1.21–2.63)	1.41 (1.03–1.95)	1.37 (0.98–2.10)	0.94 (0.69–1.28)	0.96 (0.67–1.42)

* cut off at the 75th percentile, ** Adjusted for gender, age and baseline harassement, threats or violence.

**Table 5:** Associations between emotional demands at work and harassment, threats and violence stratified by level of influence over own work.

**High influence**			
	Harassment *	Threats *	Violence *
	OR	OR	OR
Emotional demands *			
low (ref)	1	1	1
high	1.53 (1.02–2.30)	1.53 (1.01–2.32)	1.62 (1.06–2.51)
Low influence *			
	Harassment *	Threats *	Violence *
	OR	OR	OR
Emotional demands *			
low (ref)	1	1	1
high	1.47 (0.64–3.36)	2.04 (0.85–4.99)	1.18 (0.48–2.88)

All analyses adjusted for gender, age and baseline and harassement, threats or violence. * cut off at the 75th percentile.

## Data Availability

The datasets used and/or analysed during the current study are available from the authors on reasonable request.
